# Chiropractic and Spinal Manipulation: A Review of Research Trends, Evidence Gaps, and Guideline Recommendations

**DOI:** 10.3390/jcm13195668

**Published:** 2024-09-24

**Authors:** Robert J. Trager, Geronimo Bejarano, Romeo-Paolo T. Perfecto, Elizabeth R. Blackwood, Christine M. Goertz

**Affiliations:** 1 Department of Orthopaedic Surgery, Duke University School of Medicine, Durham, NC 27710, USA; romeo-paolo.perfecto@duke.edu; 2 Department of Family Medicine and Community Health, School of Medicine, Case Western Reserve University, Cleveland, OH 44106, USA; 3 Connor Whole Health, University Hospitals Cleveland Medical Center, Cleveland, OH 44106, USA; 4 Department of Health Services, Policy and Practice, Brown University, Providence, RI 02912, USA; geronimo_bejarano_cardenas@brown.edu; 5 Duke University Medical Center Library & Archives, Durham, NC 27710, USA; elizabeth.blackwood@duke.edu; 6 Duke Clinical Research Institute, Duke University School of Medicine, Durham, NC 27701, USA; 7 Robert J. Margolis, MD, Center for Health Policy, Duke University, Durham, NC 27705, USA

**Keywords:** chiropractic, spinal manipulation, low back pain, bibliometrics, clinical practice guidelines, review

## Abstract

Chiropractors diagnose and manage musculoskeletal disorders, commonly using spinal manipulative therapy (SMT). Over the past half-century, the chiropractic profession has seen increased utilization in the United States following Medicare authorization for payment of chiropractic SMT in 1972. We reviewed chiropractic research trends since that year and recent clinical practice guideline (CPG) recommendations regarding SMT. We searched Scopus for articles associated with chiropractic (spanning 1972–2024), analyzing publication trends and keywords, and searched PubMed, Scopus, and Web of Science for CPGs addressing SMT use (spanning 2013–2024). We identified 6286 articles on chiropractic. The rate of publication trended upward. Keywords initially related to historical evolution, scope of practice, medicolegal, and regulatory aspects evolved to include randomized controlled trials and systematic reviews. We identified 33 CPGs, providing a total of 59 SMT-related recommendations. The recommendations primarily targeted low back pain (n = 21) and neck pain (n = 14); of these, 90% favored SMT for low back pain while 100% favored SMT for neck pain. Recent CPG recommendations favored SMT for tension-type and cervicogenic headaches. There has been substantial growth in the number and quality of chiropractic research articles over the past 50 years, resulting in multiple CPG recommendations favoring SMT. These findings reinforce the utility of SMT for spine-related disorders.

## 1. Introduction

Chiropractic is a health care profession that focuses on the diagnosis and management of musculoskeletal disorders, with an emphasis on those affecting the spine [1]. In the US, chiropractors are often the first clinicians seen for neck pain and low back pain (LBP) [2,3], and most often use spinal manipulative therapy (SMT) to address these conditions [4]. The use of chiropractic services has steadily increased in the United States (US), rising from a 12-month prevalence rate of approximately 4% in 1980 to 7% in 2002, and most recently 11% in 2022 [5,6,7].

Since the 1970s, the chiropractic profession has seen a dramatic transformation both internally and with respect to its place in the health care landscape. Internally, a stronger foundation in chiropractic educational standards and increased rigor of national board examinations were instituted in the 1960s through the 1970s [1,8]. This was followed by an increase in the quantity and quality of chiropractic research, with studies primarily focused on examining the hallmark intervention of SMT [1]. These efforts were accelerated in 1986 when the US Supreme Court upheld a lower-court decision to protect the chiropractic profession against elimination by the American Medical Association [9]. A byproduct of this forward progress was the development of the first clinical practice guideline (CPG) to recommend SMT for LBP, authored by the US Agency for Health Care Policy and Research in 1994 [10].

The authorization of Medicare coverage for chiropractic SMT in 1972 was a major milestone in chiropractic history [11,12]. This government-funded health insurer for older adults and younger people with disabilities often sets a precedent for other payers, including Medicaid and commercial insurance. Accordingly, from the 1970s through the 1990s, laws mandating commercial coverage of chiropractic care also increased [8]. New chiropractic schools have been established over the past 10 years [13], with plans to open a chiropractic educational program at the University of Pittsburgh in 2025, the first such program to be embedded within a large public university [14]. In addition, there are more options for postgraduate education programs (i.e., residencies, fellowships, and board certifications) available for doctors of chiropractic [15]. In the US, as of 2019, five percent of chiropractors practice within integrative or hospital-based departments [16] and are in increasing demand in these settings [17]. Moreover, a growing number of chiropractors are actively conducting research in integrative settings in the US [18].

Given the evolving landscape of the chiropractic profession, we aimed to assess trends in research since the authorization of Medicare coverage in 1972 and the current state of SMT-related clinical practice guideline (CPG) recommendations from 2013 to 2024 to (1) identify gaps between the current state of the science and insurance coverage policies and (2) inform future research agendas.

## 2. Materials and Methods

This review used a state-of-the-art approach to synthesize chiropractic research from 1972 through 11 March 2024 [19]. This narrative review incorporated elements of bibliometric analysis to identify publication trends and keywords related to chiropractic research and practice [20], evidence mapping to identify trends and gaps in CPGs [21], and a qualitative analysis to highlight major themes and future directions. Search strategies were developed and conducted by a professional medical librarian (EB) in consultation with the author team and included a mix of keywords and subject headings representing SMT and guidelines using a validated guideline filter [22]. Complete reproducible search strategies, including search filters, for all databases are detailed in Supplemental File S1. All citations were imported into Covidence (Covidence systematic review software, Veritas Health Innovation, Melbourne, Australia, 2024), a systematic review screening software, which also de-duplicated the citations. All searches were executed on 14 March 2024. This review article is based exclusively on the analysis of previously published literature. It does not involve any primary research with human participants, animal subjects, or medical record review. Consequently, this work did not require approval from an institutional review board or ethics committee.

To examine publication trends, we searched Scopus for journal articles and reviews with a publication date from 1972 onwards having chiropract* or chiroprax* within the title, abstract, and/or keywords. The search limiters excluded gray literature (i.e., non-traditional publications such as books, book chapters, conference material, editorials, errata, notes, and press releases) and animal research using filters related to animals and/or veterinary publications (Supplemental File S1).

To identify CPGs, searches were conducted in MEDLINE via PubMed, Embase via Elsevier, Web of Science via Clarivate, and CINAHL via EBSCOhost (Supplemental File S1). We required a publication date of at least 2013 to fulfill our objective of examining more current, up-to-date CPGs. We included CPGs described as consensus statements/guidelines, practice guidelines, or similar terminology [23]. Such guidelines were required to be applicable to any population receiving SMT. Guidelines limited to osteopathic manipulation, manipulation under anesthesia, those not written in English, and gray literature were excluded. CPGs were considered that provided recommendations regarding the appropriateness of SMT for a specific condition rather than guidelines regarding the methods of application of SMT. We identified additional CPGs by tracking citations of included articles.

We used Scopus to examine research trends and keywords due to its broad, interdisciplinary coverage and rich keyword indexing, which was used to analyze trends and create visuals [24]. Test searches revealed a greater number of articles with Scopus compared to PubMed or Web of Science. Additionally, this decision was made for logistical reasons. Scopus data can be exported and uploaded to the bibliometric software with minimal manual data cleaning. Furthermore, it is best practice to compare proprietary data from Scopus within the given database rather than across multiple databases [24,25]. In contrast, our search for CPGs required a more comprehensive approach, using multiple databases with the aim of capturing all relevant guidelines. While the research trend and keyword analyses were broad and descriptive, the CPG search results were used for focused evidence mapping, which necessitated a more rigorous and inclusive strategy to minimize the risk of missing relevant recommendations.

Relevant results were compiled in Covidence [26] and de-duplicated, with the screening of titles/abstracts and full texts performed in duplicate (RT and RP), and data extraction performed by a single author (RT) and verified by a second (GB). Data extracted from CPGs included the author’s surname, year, condition, and recommendation(s). The CPG recommendations were extracted according to a simplified scheme as follows: “Yes” was indicated when SMT was recommended as a viable stand-alone treatment option, regardless of strength of evidence; “Multimodal” when SMT was recommended to be used alongside at least one other therapy such as usual care or exercise; “No” when there was an explicit statement to avoid SMT for the condition altogether; and “Insufficient evidence” when a recommendation could not be derived due to a lack of evidence. The former two recommendations were described as “In favor,” while the latter two were described as “Not in favor”.

We used R (version 4.2.2, Vienna, Austria [27]) along with packages including ggplot2 [28] and tidyr [29] to analyze data extracted from the primary search file exported from Scopus to examine publications per year and cumulative publications. We used the R packages bibliometrix and biblioshiny [30] to import data from our Scopus search and analyze keyword trends over time. Raw search results were imported to bibliometrix, which converted them to a bibliographic data frame and cleaned the text to a consistent format. We used VOSviewer (version 1.6.20) [31] to summarize additional keywords and their interconnections visually. Via R, we used ggplot2 and dplyr [28,32] to plot a timeline of CPG recommendations.

## 3. Results

### 3.1. Publication Trends

**Figure 1:**
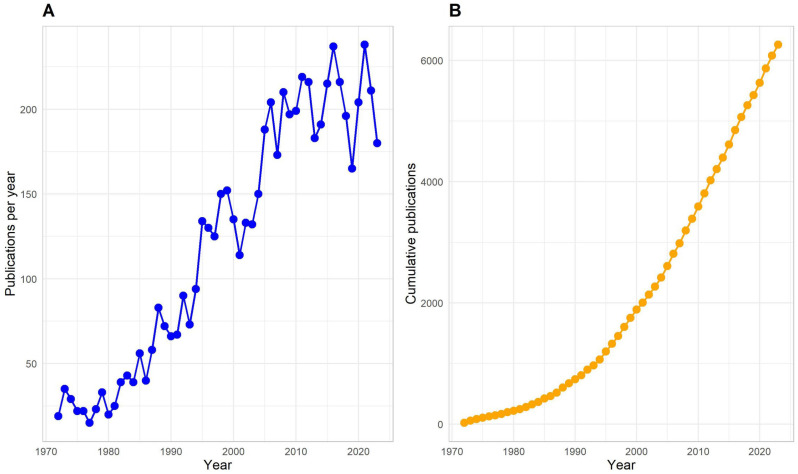
Publication trends of chiropractic research from 1970 to 2023. Image (**A**) (publications per year; blue) and Image (**B**) (cumulative publications; orange). The most recent year (2024) is not shown to provide a more accurate representation of publication trends and account for lags in indexing.

The general Scopus search identified 6286 chiropractic articles between 1972 and 2024. Few chiropractic research articles were published per year in the early 1970s; however, the rate of publications increased until the 2010s, then remained relatively static until 2023 (Figure 1).

### 3.2. Keywords

**Figure 2:**
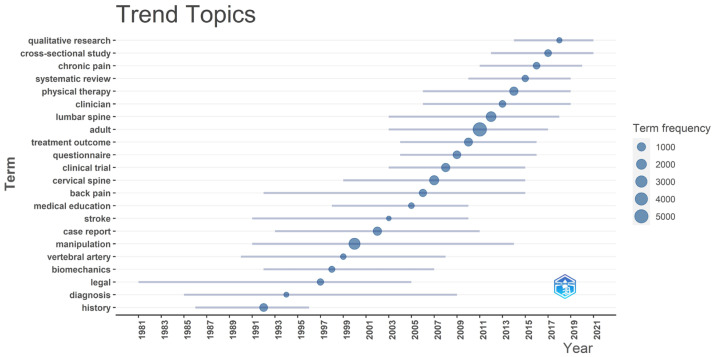
Trends in topic keywords per year from 1972 to 2024. The plot displays keywords having a frequency of ≥150 and words per year of 1, thereby tending to omit keywords from 1972 to 1981 given the relative lack of research during that time. The bars represent the first and third quartiles of keyword representation, while the circle position represents the median year of occurrence. The size of the circle corresponds to the total frequency of the keyword occurrence. We used a thesaurus to merge synonymous terms, and we removed meaningless terms (e.g., “article” and “research”) given the threshold for occurrence. The figure was created by Robert Trager using Bibliometrix and Biblioshiny.

Analysis of the trends in keywords over time revealed distinct patterns in research designs, populations, and topics of focus (Figure 2). Prior to the year 2000, the common keywords included “history”, “diagnosis”, and “legal”. This is accounted for by the corpus of articles focusing on the historical evolution, scope of practice, medicolegal, and regulatory aspects of chiropractic [33,34]. Around the year 2000, a new theme of keywords emerged, including “vertebral artery”, “manipulation”, “case report”, and “stroke”, likely resulting from numerous case reports published during this time suggesting an association between SMT and adverse vascular events.

Additional keywords emerging from the early 2000s to the mid-2010s highlighted the growth of randomized controlled trials (RCTs) examining the efficacy of SMT for back pain in adults. Common keywords included “adult”, “clinical trial”, “treatment outcome”, “manipulation”, and “back pain”. A 2019 systematic review on the topic included 47 RCTs, with only one that predated 2000 [35]. Keywords from 2015 to 2024 evolved to include “qualitative research”, “cross-sectional study”, “chronic pain”, and “systematic review”. Certain keywords were commonly represented over more than one decade, such as “back pain” (1992–2015), “lumbar spine” (2003–2018), and “cervical spine” (1999–2015).

**Figure 3:**
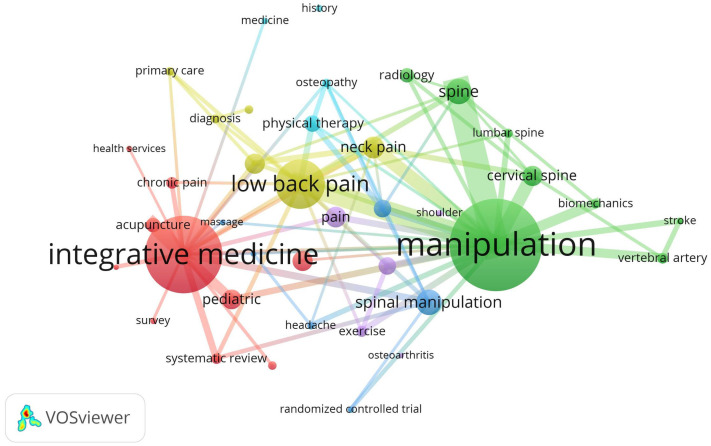
Keyword co-occurrence map (1972–2024). The figure was created using author keywords using Scopus, with the parameters of occurrences (~35), ignoring the term “chiropractic”, scale of 2.0, strength ≥ 8, using a thesaurus to merge similar terms. The size of the circles increases with greater occurrence of keyword use, while bars connecting circles indicate co-occurring keywords, with the width of bars indicating the strength of co-occurrence. The figure was created by Robert Trager using VOSviewer, version 1.6.20.

Analysis of keyword co-occurrence demonstrated six clusters centered around the following terms: “manipulation”, “pain”, “integrative medicine”, “manual therapy”, “low back pain”, and “physical therapy” (Figure 3). Regarding the two most occurring terms, “manipulation” tended to co-occur with terms related to the spine (e.g., “lumbar” or “cervical spine”) or vascular conditions (i.e., “vertebral artery” and “stroke”). In contrast, “integrative medicine” often co-occurred with a variety of terms (i.e., “pediatric”, “chronic pain”, and “acupuncture”).

### 3.3. Clinical Practice Guidelines

**Figure 4:**
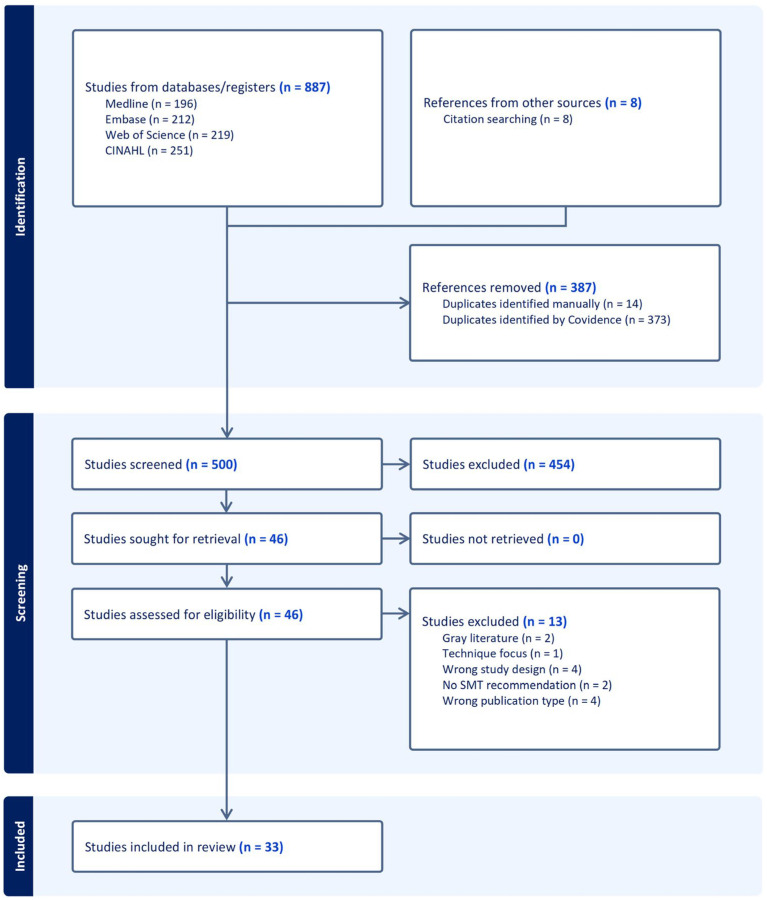
Article selection diagram. Abbreviations: Cumulative Index to Nursing and Allied Health Literature (CINAHL), spinal manipulative therapy (SMT). Please note that a large number of studies were excluded at the title/abstract phase due to our broad search strategy. Many articles mentioned clinical practice guidelines but did not constitute guidelines themselves (e.g., protocols, randomized trials, guideline adherence studies). Full-text exclusions with specific reasons are detailed in Supplemental File S2.

The CPG search identified 500 unique citations (Figure 4). For title/abstract screening, reviewers had 74% agreement (Cohen’s kappa = 0.23 [fair]), and for full-text screening, they had 93% agreement (Cohen’s kappa = 0.85 [almost perfect]). We included 33 CPGs, providing a total of 59 distinct recommendations, as several guidelines provided more than one recommendation [36,37,38,39,40,41,42,43,44,45,46,47,48,49,50,51,52,53,54,55,56,57,58,59,60,61,62,63,64,65,66,67,68] (Table 1). A list of citations excluded at the full-text phase is available in Supplemental File S2. A single CPG focused exclusively on pediatric patients [52], while another single CPG focused on older adults [48]. When explicitly described, all CPG recommendations focused on individuals with the absence of serious pathology (e.g., cancer, infection, fracture). Several CPGs also outlined red flag indicators of possible serious pathology (e.g., history of malignancy) that would prompt additional evaluation or preclude SMT altogether [38,39,41,44,46,47,48,49,50,52,54,57,59,66,67,68,69].

**Figure 5:**
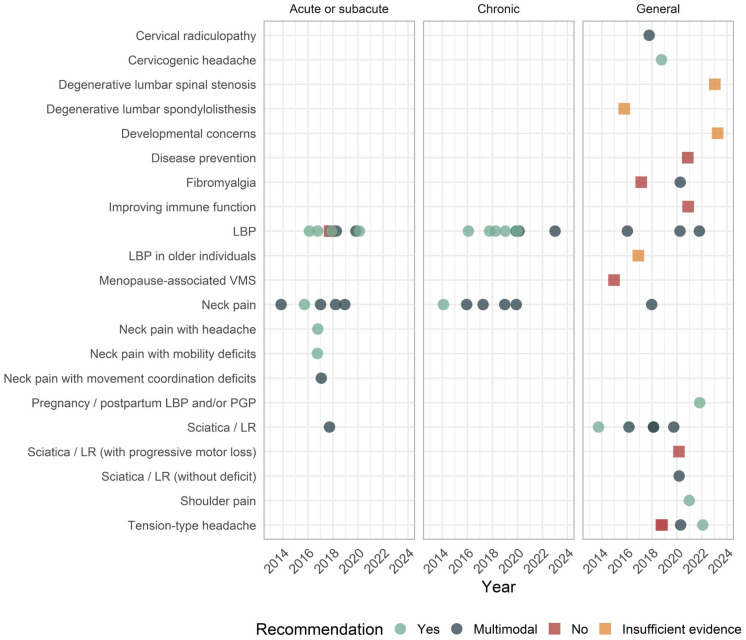
Timeline plot of clinical practice guideline recommendations for spinal manipulation by condition and year from 2014 to 2024. Recommendations for episodic and chronic tension-type headaches were grouped under “Any” to simplify the plot, considering these terms typically denote headache frequency rather than duration. Abbreviations: lower back pain (LBP), lumbar radiculopathy (LR), pelvic girdle pain (PGP), vasomotor symptoms (VMS). Recommendations in favor (“Yes” or “Multimodal”) are shown in light and dark green, respectively, while recommendations not in favor (“No” or “Insufficient evidence”) are shown in red and orange, respectively.

SMT was most often recommended for LBP (n = 19), neck pain (n = 14), sciatica/lumbar radiculopathy (n = 6), and tension-type headaches (n = 2). The use of SMT for LBP was favored in 90% of statements, while the use of SMT for neck pain was favored in 100% of statements. More recent recommendations favored the use of SMT for pregnancy and postpartum-related LBP (in 2022), cervical radiculopathy (2018), cervicogenic headaches (2019), fibromyalgia (2020), tension-type headaches (2020 and 2022), and shoulder pain (2021), in some cases replacing older recommendations that advised against it for fibromyalgia and tension-type headaches. SMT was specifically not recommended for eight conditions, including degenerative lumbar stenosis and spondylolisthesis, and developmental concerns in children (Figure 5).

Clinical practice guideline recommendations regarding SMT were further categorized based on the duration of symptoms. Accordingly, 100% (8/8) of recommendations for chronic LBP favored SMT, 88% (7/8) for acute/subacute LBP favored SMT, and 80% (4/5) of those for general LBP (regardless of duration) favored SMT. All recommendations for acute/subacute (8/8), chronic (5/5), and general neck pain (1/1) favored SMT (i.e., 100% each; Table 1 and Figure 5).

## 4. Discussion

This review found that chiropractic research has focused primarily on the use of SMT for LBP, with a recent emphasis on evidence synthesis and observational studies. While CPG recommendations have evolved, we found that recommendations in favor of SMT for LBP have been established for several years. In addition, there is both a growing interest in the chiropractic management of special populations (e.g., pregnant women, older adults) [48,65] and growing support for the use of SMT in the management of conditions not confined to the spine (e.g., chronic pain, tension-type and cervicogenic headaches, and shoulder pain) [45,49,68].

We identified a progression from case reports to larger and more rigorous study designs, including clinical trials and systematic reviews. For example, consistent with a previous bibliometric study focused on this topic [70], we found a decline over time in the publication of case reports focused on adverse events following chiropractic SMT. Terms such as “legal”, “vertebral artery”, and “stroke” diminished in frequency towards 2010, a trend that has continued as new studies show no definitive causal association between SMT and adverse vascular events such as cervical arterial dissection [71,72,73]. It is estimated that serious adverse events, such as fractures, are rare and occur between 1 per 2 million manipulations and 13 per 10,000 patients [74,75]. Likely as a result, SMT is increasingly recommended by CPGs for neck pain [38,39,40,42,43,49,53,66], with some CPGs taking into account the low risk of adverse events when deriving statements favoring SMT [40,42,53].

The recent appearance of “cross-sectional” and “qualitative” study keywords suggests that observational study designs may be increasing in frequency. Recent large observational studies have examined the association between initial provider type and downstream health service utilization among adults with LBP, showing that receiving chiropractic care is associated with a reduced likelihood of costly procedures and greater CPG adherence with respect to medication utilization [76,77,78,79,80]. These designs have several attractive features to examine non-pharmacologic interventions used by chiropractors, including high feasibility, low cost, and applicability to health service utilization and adverse events [81]. Additional studies are needed to further explore markers of care effectiveness and corroborate the already-existing CPG recommendations for chiropractic care among those with LBP. Researchers may also consider using causal inference methods within observational studies, such as instrumental variables and difference-in-differences, to better control for confounding variables and strengthen the studies’ validity [82].

We found that the number of CPGs recommending the use of SMT has increased across a growing number of musculoskeletal conditions. Most CPG statements that recommended SMT focused on LBP and neck pain either in isolation or as part of a multimodal treatment approach. While most CPGs that considered SMT recommended in favor of its use for spine-related conditions of LBP (90%) and neck pain (100%), three CPG statements found insufficient evidence to recommend SMT for degenerative lumbar spinal stenosis and spondylolisthesis [51,56,60]. Furthermore, although SMT for sacroiliac joint pain is commonly used by doctors of chiropractic and there is emerging evidence of efficacy [83], we did not identify any current CPGs that addressed the use of SMT for this condition. Such gaps suggest that additional RCTs focusing on individual subsets of LBP and distinct populations may be warranted.

The keyword “headache” was one of the most commonly identified in our search. However, it was only recently that CPGs recommended SMT for both cervicogenic and tension-type headaches (2019–2022) [44,45,49]. This early interest and subsequent CPG adoption may be explained by the fact that neck pain, commonly treated by chiropractors, is present in approximately 90% of patients with these types of headaches [84,85]. Of note, “migraine” did not appear in either keyword trends or CPG recommendations despite being one of the most prevalent headache subtypes and often treated by chiropractors [86]. A meta-analysis published in 2020 found that SMT may be effective in reducing pain intensity and days with pain for those with migraine headaches, yet concluded that more research is needed [87]. A larger randomized controlled trial examining the efficacy of SMT for migraines is currently underway [88].

We found only a single CPG dedicated to pediatric patients, although “pediatric” was a common keyword [52]. Most CPG recommendations favoring SMT for LBP generally applied to all adults, including older adults. However, one CPG, published in 2017, found insufficient evidence for treating LBP in adults aged 65 and older, related to a limited number of primary studies dedicated to this population [48]. While age-related physiological changes (e.g., reduced bone mineral density and flexibility) were postulated to play a role in how SMT is used when considering older adults with LBP [48], it remains unclear whether the efficacy of SMT differs in this demographic. In addition, the literature regarding SMT for older adults has continued to grow since the 2017 CPG. Illustratively, a recent clinical trial showed promise with regards to the effectiveness of SMT for lumbar stenosis in older patients [89]. Accordingly, it remains unclear whether future CPG statements would reach a similar conclusion.

This narrative review has several strengths, including the use of a comprehensive search strategy to identify CPGs, the incorporation of multiple data-driven and quantitative strategies to inform our qualitative interpretation of the literature, and the use of manual data extraction for CPG recommendations.

Several limitations should be considered. While our CPG search was comprehensive, our search using Scopus to examine research trends and keywords may have yielded different results if conducted using another database. For CPG recommendations, it was outside of the scope of our study to grade the strength of recommendations or quality of evidence given the large number of synthesized articles, the challenge of reconciling differences in the presentation of CPG statements, and the consideration that not all CPGs quantified or specified a strength of recommendation. Clinicians should refer to the original CPGs for further guidance on the nuances of specific clinical presentations for each condition. Our choice to focus on CPG recommendations for SMT precluded our ability to examine a broader range of therapies that chiropractors may use, such as soft tissue therapies and exercise [4].

## 5. Conclusions

Most chiropractic research articles and CPGs regarding SMT have focused on spinal pain in adults. From 1972 to 2024, research has transitioned from legal topics and case reports to randomized trials, observational studies, and evidence synthesis. We also found that there has been substantial growth in the number and rigor of standard scientific methods of chiropractic research articles over the past 50 years, resulting in multiple CPG recommendations favoring SMT. These findings reinforce the clinical utility of SMT for spine-related disorders. Additional high-quality research followed by revised CPG development is needed for understudied conditions.

## Figures and Tables

**Table 1:** Clinical practice guideline recommendations for spinal manipulative therapy.

Condition/Author	Year	Timing	Recommendation
**Cervical radiculopathy**			
Chou	2018	NA	In favor: multimodal
**Cervicogenic headache**			
Côté	2019	NA	In favor
**Degenerative lumbar spinal stenosis**			
Kawakami	2023	NA	Insufficient evidence
Kreiner	2013	NA	Insufficient evidence
**Degenerative lumbar spondylolisthesis**			
Matz	2016	NA	Insufficient evidence
**Developmental concerns**			
Keating	2023	NA	Insufficient evidence
**Disease prevention**			
Hawk	2021	NA	Not in favor
**Fibromyalgia**			
Hawk	2020	NA	In favor: multimodal
Macfarlane	2017	NA	Not in favor
**Improving immune function**			
Hawk	2021	NA	Not in favor
**Low back pain**			
Alperovitch-Najenson	2023	Chronic	In favor: multimodal
Bussières	2018	Acute or subacute	Not in favor
Bussières	2018	Chronic	In favor
Chou	2018	Acute	In favor: multimodal
Globe	2016	Acute	In favor
Globe	2016	Chronic	In favor
Hawk	2020	Chronic	In favor: multimodal
Hegmann	2020	Acute	In favor: multimodal
Hegmann	2020	Chronic	In favor: multimodal
Kreiner	2020	Acute	In favor
Kreiner	2020	Chronic	In favor
Lisi	2018	Acute or subacute	In favor
Lisi	2018	Chronic	In favor
Ma	2019	Chronic	In favor
NICE (UK)	2016	NA	In favor: multimodal
NICE (UK)	2020	NA	In favor: multimodal
Qaseem	2017	Acute or subacute	In favor
Stochkendahl	2018	Acute or subacute	In favor: multimodal
Whalen	2022	NA	In favor: multimodal
**LBP in older individuals**			
Hawk	2017	NA	Insufficient evidence
**Menopause-associated vasomotor symptoms**			
Carpenter	2015	NA	Not in favor
**Neck pain**			
Bier	2018	NA	In favor: multimodal
Blanpied	2017	Chronic	In favor: multimodal
Bryans	2014	Acute or subacute	In favor: multimodal
Bryans	2014	Chronic	In favor
Bussières	2016	Acute or subacute	In favor
Bussières	2016	Chronic	In favor: multimodal
Chou	2018	Acute	In favor: multimodal
Hawk	2020	Chronic	In favor: multimodal
Kjaer	2017	Acute or subacute	In favor: multimodal
Whalen	2019	Acute or subacute	In favor: multimodal
Whalen	2019	Chronic	In favor: multimodal
**Neck pain with headache**			
Blanpied	2017	Subacute	In favor
**Neck pain with mobility deficits**			
Blanpied	2017	Acute or subacute	In favor
**Neck pain with movement coordination deficits**			
Blanpied	2017	Subacute	In favor: multimodal
**Pregnancy-related or postpartum LBP and/or PGP**			
Weis	2022	NA	In favor
**Sciatica/LR**			
Bussières	2018	NA	In favor: multimodal
Chou	2018	NA	In favor: multimodal
Kreiner	2014	NA	In favor
NICE (UK)	2016	NA	In favor: multimodal
NICE (UK)	2020	NA	In favor: multimodal
Stochkendahl	2018	Acute or subacute	In favor: multimodal
**Sciatica/LR (with progressive motor loss)**			
Hegmann	2020	NA	Not in favor
**Sciatica/LR (without deficit)**			
Hegmann	2020	NA	In favor: multimodal
Shoulder pain			
Yu	2021	NA	In favor
**Tension-type headache**			
Côté	2019	Chronic	Not in favor
Côté	2019	Episodic	Not in favor
Dowell	2022	NA	In favor
Hawk	2020	Chronic	In favor: multimodal

Abbreviations: recommendation applied to any timing, or timing information did not apply (NA); lumbar radiculopathy (LR); National Guideline Centre (NICE); pelvic girdle pain (PGP); United Kingdom (UK). Guidelines are grouped by condition (shown in bold) and author (not bold) in the first column.

## Data Availability

Data are contained within the article or Supplementary Materials.

## Supplementary Materials

The following supporting information can be downloaded at: https://www.mdpi.com/article/10.3390/jcm13195668/s1, File S1: Search strategies; File S2: References excluded during the full-text phase.
