# Supplementary material for: Chiropractic and Spinal Manipulation: A Review of Research Trends, Evidence Gaps, and Guideline Recommendations
Source: J Clin Med. 2024 Sep 24;13(19):5668. doi: 10.3390/jcm13195668 (PMC11476883; doi:10.3390/jcm13195668)
Supplement: Supplementary file 1 [file jcm-13-05668-s001.zip › S1 File.pdf]

Search Strategies

Librarian Searcher: Elizabeth Blackwood, MSLS; Duke University Medical Center Library & Archives, Duke University School of Medicine

Scopus (via Elsevier)
Date of Search: 3/11/2024

Concept	Strategy	Results
Pub Trends; Scopus Only	TITLE-ABS-KEY ("chiropract*" OR "chiroprax*") AND PUBYEAR > 1971 AND NOT (TITLE-ABS-KEY ("animal*") OR TITLE-ABS-KEY ("dog*") OR TITLE-ABS-KEY ("horse*") OR TITLE-ABS-KEY ("pig*") OR TITLE-ABS-KEY ("rabbit*") OR TITLE-ABS-KEY ("rat*") OR TITLE-ABS-KEY ("mouse") OR TITLE-ABS-KEY ("mice") OR TITLE-ABS-KEY ("equine*") OR TITLE-ABS-KEY ("canine*") OR TITLE-ABS-KEY ("veterinar*")) AND (LIMIT-TO (SRCTYPE , "j")) AND (LIMIT-TO (DOCTYPE , "ar") OR LIMIT-TO (DOCTYPE , "re"))	6,282

MEDLINE (via PubMed)
Date of Search: 3/11/2024

Concept	Strategy	Results
#1 Spinal Manipulation	"Manipulation, Chiropractic"[Mesh] OR "Manipulation, Spinal"[Mesh] OR "Manipulation, Osteopathic"[Mesh] OR "Manipulation, Orthopedic"[Mesh] OR "spinal manipulation"[tiab] OR chiropract*[tiab] OR chiroprax*[tiab] OR "osteopathic manipulation"[tiab] OR "orthopedic manipulation"[tiab] OR "orthopaedic manipulation"[tiab]	14,001
#2 Clinical Practice Guidelines (Narrow)	"Guideline"[pt] OR "practice guideline"[pt] OR "consensus development conference"[pt] OR "consensus development conference, NIH"[pt] OR guideline*[ti] OR standards[ti] OR consensus*[ti] OR recommendat*[ti] OR guideline*[cn] OR standards[cn] OR consensus*[cn] OR recommendat*[cn] OR "practice parameter*"[ti] OR "position statement*"[ti] OR "practice bulletin*"[ti] OR "policy statement*"[ti] OR CPG[ti] OR CPGs[ti] OR "best practice*"[ti] OR (care[ti] AND (path[ti] OR paths[ti] OR pathway[ti] OR pathways[ti] OR map[ti] OR maps[ti] OR plan[ti] OR plans[ti] OR standard[ti])) OR ((critical[ti] OR clinical[ti] OR practice[ti]) AND (path[ti] OR paths[ti] OR pathway[ti] OR pathways[ti] OR protocol*[ti])) OR (algorithm*[ti] AND (pharmacotherap*[ti] OR chemotherap*[ti] OR chemotreatment*[ti] OR therap*[ti] OR treatment*[ti] OR intervention*[ti])) OR (algorithm*[ti] AND (screening[ti] OR examination[ti] OR test[ti] OR tested[ti] OR testing[ti] OR assessment*[ti] OR diagnosis[ti] OR diagnoses[ti] OR diagnosed[ti] OR diagnosing[ti])) OR guideline*[ot] OR standards[ot] OR	305,495

	consensus*[ot] OR recommendat*[ot] OR "practice parameter*"[ot] OR "position statement*"[ot] OR "practice bulletin*"[ot] OR "policy statement*"[ot] OR CPG[ot] OR CPGs[ot] OR "best practice*"[ot] OR (care[ot] AND (path[ot] OR paths[ot] OR pathway[ot] OR pathways[ot] OR map[ot] OR maps[ot] OR plan[ot] OR plans[ot] OR standard[ot])) OR ((critical[ot] OR clinical[ot] OR practice[ot]) AND (path[ot] OR paths[ot] OR pathway[ot] OR pathways[ot] OR protocol*[ot])) OR (algorithm*[ot] AND (pharmacotherap*[ot] OR chemotherap*[ot] OR chemotreatment*[ot] OR therap*[ot] OR treatment*[ot] OR intervention*[ot])) OR (algorithm*[ot] AND (screening[ot] OR examination[ot] OR test[ot] OR tested[ot] OR testing[ot] OR assessment*[ot] OR diagnosis[ot] OR diagnoses[ot] OR diagnosed[ot] OR diagnosing[ot])) OR (("Systematic review"[ti] OR "systematic review"[pt] OR "systematic review"[ot]) AND ("practice guideline*"[tiab] OR "treatment guideline*"[tiab] OR "clinical guideline*"[tiab] OR "guideline recommendation*"[tiab]))	
#3 Combined	#1 AND #2	345
#4 Filter Animal Studies	#3 NOT (animals[MeSH Terms] NOT humans[MeSH Terms])	345
#5 Time limit	Limit to 2013, PubMED user interface	196

Embase (via Elsevier)
Date of Search: 3/11/2024

**Concept**	**Strategy**	**Results**
#1 Spinal Manipulation	'chiropractic manipulation'/exp OR 'spine manipulation'/exp OR 'osteopathic manipulation'/exp OR 'orthopedic manipulation'/exp OR ('spinal manipulation' OR chiropract* OR chiroprax* OR 'osteopathic manipulation' OR 'orthopedic manipulation' OR 'orthopaedic manipulation'):ti,ab	11,627
#2 Clinical Practice Guidelines (Narrow)	guideline*:ti OR standards:ti OR consensus*:ti OR recommendat*:ti OR guideline*:au OR standards:au OR consensus*:au OR recommendat*:au OR 'practice parameter*':ti OR 'position statement*':ti OR 'practice bulletin*':ti OR 'policy statement*':ti OR CPG:ti OR CPGs:ti OR 'best practice*':ti OR (care:ti AND (path:ti OR paths:ti OR pathway:ti OR pathways:ti OR map:ti OR maps:ti OR plan:ti OR plans:ti OR standard:ti)) OR ((critical:ti OR clinical:ti OR practice:ti) AND (path:ti OR paths:ti OR pathway:ti OR pathways:ti OR protocol*:ti)) OR (algorithm*:ti AND (pharmacotherap*:ti OR chemotherap*:ti OR chemotreatment*:ti OR therap*:ti OR treatment*:ti OR intervention*:ti)) OR (algorithm*:ti AND (screening:ti OR examination:ti OR test:ti OR tested:ti OR testing:ti OR assessment*:ti OR diagnosis:ti OR diagnoses:ti OR diagnosed:ti OR diagnosing:ti)) OR guideline*:kw OR standards:kw OR consensus*:kw OR recommendat*:kw OR 'practice parameter*':kw OR 'position statement*':kw OR 'practice bulletin*':kw OR 'policy statement*':kw	377,976

	OR CPG:kw OR CPGs:kw OR 'best practice*':kw OR (care:kw AND (path:kw OR paths:kw OR pathway:kw OR pathways:kw OR map:kw OR maps:kw OR plan:kw OR plans:kw OR standard:kw)) OR ((critical:kw OR clinical:kw OR practice:kw) AND (path:kw OR paths:kw OR pathway:kw OR pathways:kw OR protocol*:kw)) OR (algorithm*:kw AND (pharmacotherap*:kw OR chemotherap*:kw OR chemotreatment*:kw OR therap*:kw OR treatment*:kw OR intervention*:kw)) OR (algorithm*:kw AND (screening:kw OR examination:kw OR test:kw OR tested:kw OR testing:kw OR assessment*:kw OR diagnosis:kw OR diagnoses:kw OR diagnosed:kw OR diagnosing:kw)) OR (('Systematic review':ti OR term:it OR 'systematic review':kw) AND ('practice guideline*':ti,ab OR 'treatment guideline*':ti,ab OR 'clinical guideline*':ti,ab OR 'guideline recommendation*':ti,ab))	
#3 Combined	#1 AND #2	336
#4 Filter Animal Studies	#3 NOT ([animals]/lim NOT [humans]/lim)	336
#5 Date limiter	Publication years: 2013-2024	212

Web of Science (via Clarivate)
Date of Search: 3/11/2024

Concept	Strategy	Results
#1 Spinal Manipulation	TS=("spinal manipulation" OR chiropract* OR chiroprax* OR "osteopathic manipulation" OR "orthopedic manipulation" OR "orthopaedic manipulation")	7,032
#2 Clinical Practice Guidelines (Narrow)	TS=("Guideline" OR "practice guideline" OR "consensus development conference" OR "practice parameter*" OR "position statement*" OR "practice bulletin*" OR "policy statement*" OR CPG OR CPGs OR "best practice*") OR TI=((guideline* OR standards OR consensus* OR recommendat* OR "practice parameter*" OR "position statement*" OR "practice bulletin*" OR "policy statement*" OR CPG OR CPGs OR "best practice*" OR (care AND (path OR paths OR pathway OR pathways OR map OR maps OR plan OR plans OR standard)) OR ((critical OR clinical OR practice) AND (path OR paths OR pathway OR pathways OR protocol*)) OR (algorithm* AND (pharmacotherap* OR chemotherap* OR chemotreatment* OR therap* OR treatment* OR intervention*)) OR (algorithm* AND (screening OR examination OR test OR tested OR testing OR assessment* OR diagnosis OR diagnoses OR diagnosed OR diagnosing))))	723,439
#3 Combined	#1 AND #2	358
#4 Filter Animal Studies	#3 NOT TS=((animal* OR rat OR rats OR mouse OR mice OR murine OR dog OR dogs OR canine OR cat OR cats OR feline OR rabbit OR cow OR cows OR bovine OR rodent* OR sheep OR ovine OR pig OR swine OR porcine OR veterinar* OR chick* OR zebrafish* OR baboon* OR	356

	nonhuman* OR primate* OR cattle* OR goose OR geese OR duck OR macaque* OR avian* OR bird* OR fish*) NOT (human* OR patient* OR women OR woman OR men OR man))	
#5 Date limiter	Publication years: 2013-2024	219

CINAHL (via EBSCOhost)
Date of Search: 3/11/2024

Concept	Strategy	Results
S1 Spinal Manipulation	(MH "Manipulation, Chiropractic") OR (MH "Manipulation, Orthopedic") OR (MH "Manipulation, Orthopedic") OR (MH "Chiropractic+") OR (MH "Chiropractic Practice") OR (MH "Chiropractors") OR "spinal manipulation" OR chiropract* OR chiroprax* OR "osteopathic manipulation" OR "orthopedic manipulation" OR "orthopaedic manipulation"	37,438
S2 Clinical Practice Guidelines (Narrow)	PT (practice guidelines or standards or protocol or critical path or care plan) or TI (guideline* or standards or consensus* or recommendat*) or AU (guideline* or standards or consensus* or recommendat*) or CA (guideline* or standards or consensus* or recommendat*) or TI ("practice parameter*" or "position statement*" or "policy statement*" or CPG or CPGs or "best practice*") or TI (care N2 path or care N2 paths or care N2 pathway or care N2 pathways or care N2 map or care N2 maps or care N2 plan or care N2 plans or care N2 standard*) or TI (critical N2 path or critical N2 paths or critical N2 pathway or critical N2 pathways or critical N2 protocol* or clinical N2 path or clinical N2 paths or clinical N2 pathway or clinical N2 pathways or clinical N2 protocol* or practice N2 path or practice N2 paths or practice N2 pathway or practice N2 pathways or practice N2 protocol*) or TI (algorithm* AND (pharmacotherap* or chemotherap* or chemotreatment* or therap* or treatment* or intervention*)) or (PT algorithm AND TI (pharmacotherap* or chemotherap* or chemotreatment* or therap* or treatment* or intervention*)) or TI (algorithm* AND (screening or examination or test or tested or testing or assessment* or diagnosis or diagnoses or diagnosed or diagnosing)) or (PT algorithm AND TI (screening or examination or test or tested or testing or assessment* or diagnosis or diagnoses or diagnosed or diagnosing))	170,301
S3 Combined	S1 AND S2	704
S4 Filter Animal Studies	S3 NOT (((MH "Animals+") OR (MH "Animal Studies") OR (TI "animal model*")) NOT (MH "human"))	704
S5 Date Limiter	S4 Limit to 2013-2024	251
