# Supplementary material for: Chiropractic and Spinal Manipulation: A Review of Research Trends, Evidence Gaps, and Guideline Recommendations
Source: J Clin Med. 2024 Sep 24;13(19):5668. doi: 10.3390/jcm13195668 (PMC11476883; doi:10.3390/jcm13195668)
Supplement: Supplementary file 1 [file jcm-13-05668-s001.zip › S2 File.pdf]

References excluded at full-text phase

1. Bussières, A. "Canadian Chiropractic Guideline Initiative (CCGI) Progress and Future Directions: December 2017." *J Can Chiropr Assoc* 61, no. 3 (2017): 186–89.
2. ———. "The Canadian Chiropractic Guideline Initiative: Progress to Date." *J Can Chiropr Assoc* 58, no. 3 (2014): 215–19.
3. Hawk, C., M. J. Schneider, S. Vallone, and E. G. Hewitt. "Best Practices for Chiropractic Care of Children: A Consensus Update." *J Manipulative Physiol Ther* 39, no. 3 (2016): 158–68. https://doi.org/10.1016/j.jmpt.2016.02.015.
4. Kopansky-Giles, D. "Premature Evaluation–Making Policy Recommendations in the Absence of Sufficient Evidence." *Journal of Manipulative and Physiological Therapeutics* 44, no. 6 (2021): 507–8. https://doi.org/10.1016/j.jmpt.2021.04.007.
5. Melloh, M. "Management of Acute Low Back Pain through Guidelines-Wishful Thinking? An Update on Clinical Practice Guidelines on Acute Low Back Pain Management in Primary Care." *Internal Medicine Journal* 43 (2013): 33. https://doi.org/10.1111/imj.12154.
6. National Guideline, Centre. "NICE Evidence Reviews Collection." *Evidence Review for Manual Therapy for Chronic Primary Pain: Chronic Pain (Primary and Secondary) in over 16s: Assessment of All Chronic Pain and Management of Chronic Primary Pain: Evidence Review I PB - National Institute for Health and Care Excellence (NICE) Copyright © NICE 2021.*, 2021.
7. Niu, Jian-fei, Xiao-feng Zhao, Han-tong Hu, Jia-jie Wang, Yan-ling Liu, and De-hua Lu. "Should Acupuncture, Biofeedback, Massage, Qi Gong, Relaxation Therapy, Device-Guided Breathing, Yoga and Tai Chi Be Used to Reduce Blood Pressure?: Recommendations Based on High-Quality Systematic Reviews." *Complementary Therapies in Medicine* 42 (2019): 322–31. https://doi.org/10.1016/j.ctim.2018.10.017.
8. Shufelt, C. L. "2023 Nonhormone Therapy Position Statement." *Menopause* 30, no. 12 (2023): 1253–54. https://doi.org/10.1097/GME.0000000000002288.
9. "Summary of Recommendations." *Journal of Clinical Chiropractic Pediatrics* 18, no. 2 (2019): 6–7.
10. Tick, H., and A. Nielsen. "Academic Consortium for Integrative Medicine & Health Commentary to Health and Human Services (HHS) on Inter-Agency Task Force Pain Management Best Practices Draft Report." *Global Advances In Health and Medicine* 8 (2019). https://doi.org/10.1177/2164956119857656.
11. Touchette, J., A. St-Jean, D. Brousseau, L. Marcotte, J. Théroux, and C. Doucet. "Critical Appraisal for Existing Clinical Guidelines on the Detection and Conservative Care for Adolescent Idiopathic Scoliosis (AIS) Using AGREE II." *Scoliosis and Spinal Disorders* 12 (2017). https://doi.org/10.1186/s13013-017-0124-0.
12. Wong, J. J., P. Côté, D. A. Sutton, K. Randhawa, H. Yu, S. Varatharajan, R. Goldgrub, et al. "Clinical Practice Guidelines for the Noninvasive Management of Low Back Pain: A Systematic Review by the Ontario Protocol for Traffic Injury Management (OPTIMa) Collaboration." *EUROPEAN JOURNAL OF PAIN* 21, no. 2 (2017): 201–16. https://doi.org/10.1002/ejp.931.

13. Zaina, F., P. Côté, C. Cancelliere, F. Di Felice, S. Donzelli, A. Rauch, L. Verville, S. Negrini, and M. Nordin. "A Systematic Review of Clinical Practice Guidelines for Persons With Non-Specific Low Back Pain With and Without Radiculopathy: Identification of Best Evidence for Rehabilitation to Develop the WHO's Package of Interventions for Rehabilitation." *Arch Phys Med Rehabil* 104, no. 11 (2023): 1913–27. https://doi.org/10.1016/j.apmr.2023.02.022.
